# Do we need to vaccinate every child against COVID-19: What evidence suggests—A systematic review of opinions

**DOI:** 10.3389/fpubh.2022.1002992

**Published:** 2022-11-08

**Authors:** Sourabh Paul, Chandra Mauli Mishra

**Affiliations:** Department of Community and Family Medicine, All India Institute of Medical Sciences, Raebareli, Uttar Pradesh, India

**Keywords:** COVID-19, SARS-CoV-2, children, adverse events, vaccine safety, COVID vaccine, immunization

## Abstract

It is still debatable whether all children should receive the COVID-19 vaccine. The comparatively mild cases and low risk of COVID-19 in children compared to adults, as well as the lack of clarity on the relative effects of the disease and vaccine, indicate that the risk-benefit ratio of vaccination in children is more nuanced. To consider and highlight the complexity of policy decisions regarding COVID-19 vaccination in children, we outlined the points regarding for and against vaccination of children against COVID-19 in this systemic review. Using Medical Search Headings (MeSH) terms and keywords, we searched PubMed, PubMed Central, Scopus, and Google Scholar. The primary search term was COVID-19 vaccination (all synonyms), factors (all synonyms), and among children (all synonyms). A total of 367 articles were searched. Finally, 64 articles met the inclusion criteria and were included in the review. The major theme/tone of 28 (43.75%) articles was in favor of children's COVID vaccination, and they were highlighting the positive factors, whereas the major theme/tone of 20 (31.25%) articles was against it. Approximately 16 (25.0%) articles were in a neutral position. Major factors highlighted by articles in favor of childhood COVID vaccination were as follows: the increasing rate of disease burden (29 articles), prevention of interruption of academic activities of children or school reopening (24 articles), and a role in defense against COVID infection (21 articles). Major factors against childhood vaccination were as follows: mild infection among children (27 articles), ethical concerns and legal problems regarding the consent of minors (17 articles), and vaccine hesitancy among parents for childhood vaccination (11 articles). Whereas, factors of uncertainty were the role in the reduction of community transmission (19 articles), protection against MIS-C (10 articles), and defense against long COVID (7 articles). Considering all the factors of COVID-19 disease progression among children, a cautious approach will be essential before proceeding with COVID-19 vaccination in children.

## Introduction

Due to its extremely contagious nature, COVID-19 which emerged in late December 2019, quickly spread from person to person, resulting in a global pandemic (1). Wuhan, China, was the site of the disease outbreak's initial notification in December 2019. Later, on 11 March 2020, the World Health Organization (WHO) designated COVID-19 to be a pandemic illness (2). According to the most recent data, as of 02 August 2022, there were more than 6.3 million deaths globally and more than 545 million confirmed cases of COVID-19 (3). The recently discovered variant “Omicron” was classified as a variant of concern on 26 November 2021 (4). It was also reported that a variety named “IHU” with a Cameroonian origin has been found in France. Different COVID-19 vaccines have been produced over time, and vaccination campaigns are currently being conducted in all countries to reduce mortality and morbidity caused by COVID-19 infection. The majority of vaccines available now are for adults. Children account for ~1–3% percent of all confirmed COVID-19 cases. Children have fewer severe diseases and a better prognosis than adults, and deaths are exceedingly uncommon, mostly involving teenagers and those with serious underlying comorbidities. Adults have been the primary target of COVID-19 vaccination trials, and there is currently little information on the vaccine's safety in children (5). There are deficiencies in proven studies regarding the effect of vaccination on preventing the transmission of coronavirus. WHO's position statement on COVID-19 vaccination among children says that it should be country-specific considering the epidemiological and social context (6). Whereas, the CDC suggests that children as young as 6 months to 4 years of age also should get the primary doses of the COVID-19 vaccine (7). Even GAVI has favored childhood COVID vaccination considering the poor healthcare facilities for the most vulnerable kids globally (8). The question of universal childhood COVID vaccination should be evidence-based as well as strong on the social and ethical background. Presently, there is diverse opinion and views among global experts on the issue of universal childhood COVID vaccination. In this context, the study was conducted with the following objective:

(A) To examine current expert opinions or viewpoints on universal childhood COVID vaccination.
(B) Based on expert opinion, quantify the factors associated with childhood COVID vaccination (positive/negative/uncertain).

## COVID-19 vaccines for children

The following vaccines against COVID-19 have been determined by WHO to have met the requirements for safety and efficacy in individuals aged 18 years and over as of 12 January 2022: AstraZeneca/Oxford vaccine, Johnson & Johnson, Moderna, Pfizer/BioNTech, Sinopharm, Sinovac, COVAXIN, Covovax, and Nuvaxovid. The Pfizer vaccine is safe to use for individuals 5 years of age and older, while the Moderna vaccine is safe to use for individuals 12 years of age and older, according to the Strategic Advisory Group of Experts (SAGE) of WHO (9) (Table 1).

**Table 1:** Vaccines approved for administration in children in different countries (9–15).

**Vaccines**	**Type of vaccine**	**Countries**	**Age group**
Pfizer-BioNTech*	mRNA	US, UAE, Oman and Saudi Arabia, Bahrain, Malaysia, Canada, Spain, New Zeland, France, Britain, Germany, Norway, Namibia, Costa Rica, Israel, and Czech Republic	5–11
		El Salvador	6–11
		Singapore	5+
		EU	5–18
		Japan, Jordan, Australia, Philippines, Estonia, Denmark, Greece, Ireland, Lithuania, Sweden, and Finland	12+
		Switzerland, Italy, Greece, Poland, and Austria	12–15
		Brazil, South Africa, Morocco, and Guinea	12–17
		Egypt, Lithuania, and Romania	12–18
		Thailand	5–18
		Mexico and Pakistan	15+
		Vietnam	16–17
Moderna*	mRNA	Switzerland and Greece	12–15
		Italy	12–17
Sputnik M	Adenovirus DNA	Russia	12–17
Sinovac	Inactivated	China	3–17
		Indonesia, Chile, and Ecuador	6+
		Pakistan	12+
		Zimbabwe	14+
Sinopharm	Inactivated	Argentina	3+
		Bahrain	3–11
		China and UAE	3–17
		Pakistan	12+
		Morocco	12–17
Covaxin	Inactivated	India	15–18
ZycovD	DNA plasmid-based	India	12–17
Abdala	Protein subunit	Cuba	2–10
Soberana	Conjugate	Cuba and Venezuela	2–10
Astra-Zenica	ChAdOx1-S [recombinant] vaccine	Columbia	12+
Johnson & Johnson	Viral vector	Columbia	12+
Pfizer-BioNTech*	mRNA	US, UAE, Oman and Saudi Arabia, Bahrain, Malaysia, Canada, Spain, New Zeland, France, Britain, Germany, Norway, Namibia, Costa Rica, Israel, and Czech Republic	5–11
		El Salvador	6–11
		Singapur	5+
		EU	5–18
		Japan, Jordan, Australia, Philippines, Estonia, Denmark, Greece, Ireland, Lithuania, Sweden, and Finland	12+
		Switzerland, Italy, Greece, Poland, and Austria	12–15
		Brazil, South Africa, Morocco, and Guinea	12–17
		Egypt, Lithuania, and Romania	12–18
		Thailand	5–18
		Mexico and Pakistan	15+
		Vietnam	16–17
Moderna*	mRNA	Switzerland and Greece	12–15
		Italy	12–17
Sinovac	Inactivated	China	3–17
		Indonesia, Chile, and Ecuador	6+
		Pakistan	12+
		Zimbabwe	14+
Sinopharm	Inactivated	Argentina	3+
		Bahrain	3–11
		China and UAE	3–17
		Pakistan	12+
		Morocco	12–17
Covaxin	Inactivated	India	15–18
ZycovD	DNA plasmid-based	India	12–17
Abdala	Protein subunit	Cuba	2–10
Soberana	Conjugate	Cuba and Venezuela	2–10
Astra-Zenica	ChAdOx1-S [recombinant] vaccine	Columbia	12+

*Approved by WHO for children.

## Materials and methods

### Eligibility criteria

A systematic review of the articles was conducted between January 2020 and March 2022 to identify the articles discussing the factors related to COVID vaccination for children. Any factor which is directly or indirectly related to or affects the decision of COVID-19 universal vaccination among children was considered for inclusion in the study.

Some of the major probable factors considered were:

(a) Scientific knowledge about the COVID vaccine's content, efficacy, utility, and so on in childhood vaccination
(b) Programmatic aspects of childhood COVID vaccination
(c) Ethical and social factors, and so on.

The inclusion and exclusion criteria used for the inclusion and exclusion of the article identified from the search are mentioned in Table 2.

**Table 2:** Inclusion and exclusion criteria of the articles.

**Inclusion criteria**	**Exclusion criteria**
Relevant available Article	The original article was excluded.
Published in English	A study conducted or published outside the time period mentioned in the inclusion criteria is excluded
Content related to the discussion of factors related to COVID vaccination among children	The article is currently unavailable in English.
Full-text articles published in peer-reviewed journals	The content is not directly related to the study objective.
Types of articles included: Commentary, review articles, perspectives, reports, letters to the editor, etc.	Full-text article not available

### Search strategy and information source

The systematic strategy was used for literature searching using electronic databases and supplemented by hand searching and cross-referencing. A further search was also conducted on the Internet *via* search engines such as Google Scholar, and so on. We searched Pub Med, Pub Med Central, and Scopus electronic databases using the Medical Search Headings (MeSH) terms and keywords. The primary search terms were COVID-19 vaccination (all synonyms), factors (all synonyms), and among children (all synonyms). We used “text word searching.” In this “text word searching” method, we searched for the above-mentioned words' appearance anywhere in the document, not only in the full text of the article. MeSH searching was (COVID-19 vaccination OR Coronavirus immunization OR SARS-CoV-2) AND (factors OR conditions) AND (related with OR associated with) AND (among Children OR Adolescent OR Child). The articles which were included in the systematic review were cross-checked through their references and citations to confirm that all relevant articles were included.

### Selection process

**Figure 1:**
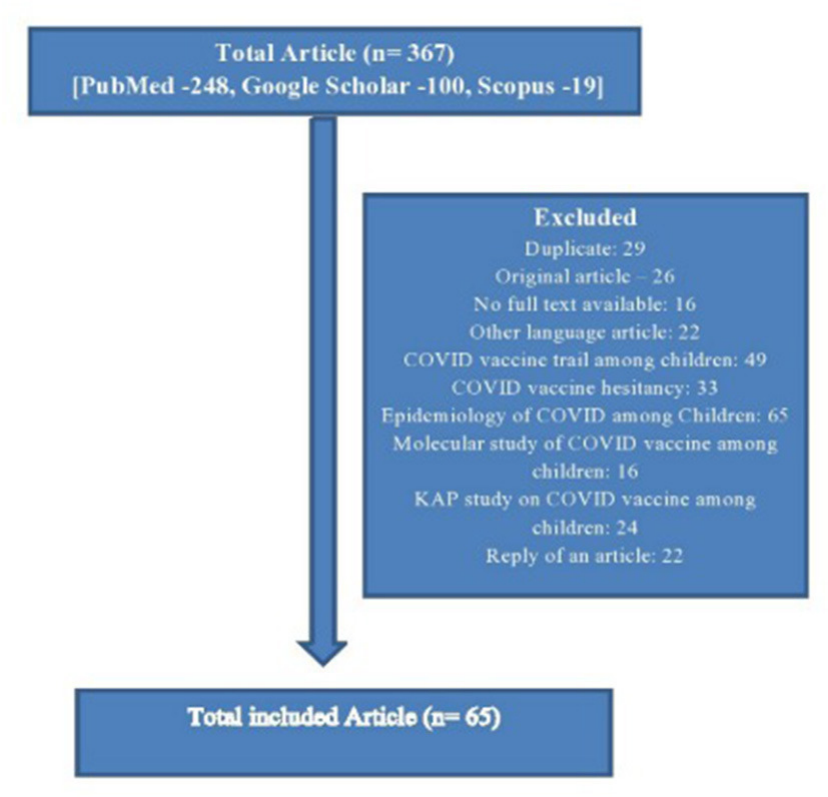
Flow diagram of the review process.

The articles that emerged from the database were screened in a two-stage process. First, the title and abstract of the study were assessed to determine whether they met the inclusion criteria. In the second stage, the full text of the included article was reviewed against the inclusion criteria. When there was any uncertainty about the inclusion or exclusion of an article, the whole text of the article was reviewed separately by two reviewers (SP and CMM) and conciseness was reached through discussion. The flow diagram which has been used for the selection of the studies is given below in Figure 1.

### The data extraction process

A standard proforma was used for data extraction.

1. Identification (article title, authors, and year of publication).
2. All the included articles were screened by each researcher individually to find out the probable factors discussed in the article about childhood COVID vaccination, and the factors were listed in the data extraction form. The only mention of a factor in an article does not qualify to include it as a factor discussed in that article. There should be a substantial discussion of that factor in that article to include it in the final data extraction form.
3. Finally, both authors identified the article's major conclusion or major theme or tone (In favor of Childhood COVID-19 vaccinations/against/both). The article's tone was derived from the factors discussed in the article. If there was more than one theme or conclusion, then the article was assigned a positive or negative based on the number of positive and negative factors discussed in it. In cases where there is uncertainty about the number of positive and negative factors, the theme or tone of the article was assigned as an uncertain or neutral category.

### Data synthesis

Initial screening of the article according to inclusion and exclusion criteria was conducted by the first author (SP), and the discrepancy was addressed by the first and second authors (SP and CMM). Data extraction of the included studies was done by the second author (CMM), and the random sample was independently checked by all others. All the disagreements were resolved by discussion.

## Results

The total number of articles searched was 367. Among them, 64 articles' were finally included according to inclusion criteria. In that, the major tone of 28 (43.75%) articles was in favor of children's COVID vaccination and they were highlighting mostly the positive factors, whereas the major themes of 20 (31.25%) articles were against it, and similarly, they were highlighting major negative factors. Approximately, 16 (25.0%) articles were in the neutral position, highlighting both the pros and cons of COVID-19 vaccination for children. According to the type of the articles, the majority were editorials (14, 21.9%), commentary or viewpoints (18, 28.1%), review articles or perspectives (11, 17.2%), letters to the editor (6, 9.4%), and others (15, 23.4%) (Table 3).

**Table 3:** Summary of the included study and description of factors discussed related to childhood COVID vaccination.

**Sr. No**.	**Title**	**Author**	**Type of article**	**Positive factors**	**Negative factors**	**Uncertain or both factor**	**The theme of the article (in favor/against/both or uncertain) for child COVID vaccination**
1	Should children be vaccinated against COVID-19? (16)	Petra Zimmermann et al. (2021)	Review	1. High disease burden 2. Protection against COVID-19, 3. Protection against severe COVID-19, 4. An early return to school 5. Positive impact in preventing the formation of new variants 6. Restoring activity and economic stability to pre-pandemic levels 7. Favor cost-effectiveness ratio 8. Reduce indirect harm of lockdown 9. Benefits of international travel	1. Children typically have milder COVID-19 infections, 2. Long-term safety unknown 3. Limited vaccine supply 4. Direct high cost of the vaccine 5. Affects the program for regular vaccinations negatively	1. Long-term safety, and efficacy unknown 2. Uncertain risk of adverse effects 3. Protection against PIMS-TS unknown 4. Defense against long COVID uncertain, 5. Unknown contribution to the decrease in disease transmission	Both or uncertain
2	The COVID-19 pandemic in children and young people during 2020-2021: A complex discussion on vaccination (17).	Igor Rudan et al. (2021)	Editorial	1. High disease burden 2. Protection against COVID-19 infection 3. School reopening and normality like the pre-pandemic period 4. Favorable risk-benefit ratio 5. Reduce fear among parents as well as children and also improve social interaction in the community 6. Social as well as an ethical responsibility 7. Prevent mutation into a new variant 8. High-risk group children will be benefited 9. Helpful in reduction of community transmission	1. Long-term safety of the vaccine unknown 2. Ethical concerns of vaccination of minors, 3. Negative effect on routine childhood immunization, 4. Vaccine hesitancy among parents	1. Unknown Safety, immunogenicity, and efficacy of vaccine among children and adolescents 2. Uncertain about adverse effects following vaccination 3. Unknown protective role against PMIS/MIS-C 4. Uncertain defense against long COVID	Both or uncertain
3	Clinical ethics: consent for vaccination in children (18).	Dominic Wilkinson (2022)	Editorial		1. COVID vaccine-related ethical concern and difficult consent process among children 2. Vaccine hesitancy among parents		Against
4	COVID-19 mRNA vaccines in adolescents and young adults: benefit-risk discussion (19).	Megan Wallace et al. (2021)	Report	1. Good vaccine safety and efficacy 2. Less number of severe adverse events 3. Defense against COVID-19 infection 4. Defense against the severe forms of COVID-19 5. Favorable risk-benefit ratio 6. Favorable role in the reduction of household transmission	1. Disease burden is not high substantially	1. Uncertain protective role against PIMS/MIS-C 2. Unknown defense against long COVID among children	Favor
5	A focused protection vaccination strategy: why we should not target children with COVID-19 vaccination policies (20).	Alberto Giubilini et al. (2021)	Commentary		1. Low disease burden, 2. No role in defense against COVID-19 infection 3. Cost-effectiveness is not favorable 4. Altered risk-benefit ratio 5. Direct cost is also high 6. Problem of the ethical issue of minor vaccination	1. Uncertain role in the reduction of community transmission	Against
6	Should children be vaccinated against COVID-19 now? (5)	Brian Li Han Wong et al. (2021)	Viewpoint	1. Only beneficial for high-risk group children 2. Favorable role in the reduction of household transmission	1. Low disease burden, 2. Milder form of the disease 3. Vaccine efficacy, safety, and effectiveness are not beneficial 4. No role of defense against COVID-19 infection		Against
7	COVID-19 in children and the importance of COVID-19 vaccination (21)	Feng Xia Xue et al. (2021)	Editorial	1. Increasing disease burden and hospitalization rate among children, 2. Preventive role in the community and school transmission, 3. Favorable Safety and effectiveness data of the vaccine 4. Less severe adverse events 5. Good defense against COVID-19 infection 6. Favorable for inclusion in the child immunization schedule 7. Improve the mental health status of the children 8. Reduction in disruption of health care facilities			Favor
8	Reasons in favor of universal vaccination campaign against COVID-19 in the pediatric population (22).	Nicola Principi et al. (2022)	Debate	1. Increasing mortality and morbidity rates among children, 2. Favorable efficacy, safety, and tolerability of the vaccine 3. All the adverse events are minor in nature 4. Favorable protection against COVID-19 infection 5. Defense against the severe forms of COVID-19 61. Beneficial for school reopening 7. Reduce fear among parents and children		1. Uncertain role in the prevention of household transmission	Favor
9	COVID-19 vaccination for children: may be necessary for the full eradication of the disease (23).	Andrea D. Praticò (2021)	Correspondence	1. Role in prevention of “Multisystem inflammatory syndrome in children” and related death, 2. Role of vaccination to prevent community transmission	1. Less efficacy and safety trial among children		Favor
10	Should we be vaccinating children against COVID-19 in high-income countries? (24)	Grace Li et al. (2021)	Expert review	1. Helps in school reopening 2. Limited information on severe adverse effects 3. Improve the mental health status of children	1. No role in the prevention of COVID-19 infection 2. Problem of vaccine inequality in society 3. No role of children in community transmission,	1. Uncertain about high disease burden in children 2. Uncertain about the role of the vaccine in the prevention of the emergence of new variant 3. Limited information on long-time safety 4. Doubtful role of the vaccine in the prevention of PIMS-TS, 5. Doubtful role in the prevention of long COVID,	Against
11	Adolescent Consent to COVID-19 Vaccination: The Need for Law Reform (25).	Robert S. Olicket al. (2022)	Public health report		1. High level of vaccine hesitancy	1. Adolescent COVID vaccination consent and ethical problems	Against
12	Vaccinating children and adolescents against severe acute respiratory syndrome coronavirus 2 (SARS-CoV-2)—the Israeli experience (26).	Daniel Glikman et al. (2021)	Editorial	1. High disease burden 2. School reopening, 3. Good effectiveness and efficacy trial results, 4. Less number of adverse events. 5. Good defense against COVID-19 infection 6. Favorable risk-benefit ratio for children 7. Well-developed infrastructure for routine immunization can be beneficial 8. Defense against long COVID-19	1. Mild form of the disease among children		Favor
13	Providing children with COVID-19 vaccinations is challenging due to the lack of data and wide-ranging parental acceptance (27).	Jiatong She et al. (2021)	Review article	1.Increasing disease burden among children 2.Vaccines is having good effectiveness, safety, and efficacy, 3.Fewer adverse events 4.Good defense against COVID-19 infection 5.Easy for school reopening 6.Faster return of the economy to prepandemic activity 7.Favorable risk-benefit ratio 8.Children's vaccination will enhance the possibility of herd immunity 9.Will improve social interaction among children 10.Stop emergence of new variant 11.Indirect benefits of vaccination for the protection of elderly 12.Reduce disruption of health care services	1.Low rate of hospitalization and death, 2.High vaccine hesitancy among parents		Favor
14	Vaccinating children against COVID-19 (28)	Jennie S. Lavine et al. (2021)	Editorial	1.Beneficial for the high-risk group of children	1.Less disease burden, 2.Mild form of infection 3.Cost-benefit is less, 4.No role of the vaccine in the prevention of disease transmission, 5.Less duration of protection, 6.An epidemiological shift in the age group of disease, 7.Possibility of high case fatality and low rate of infection among children in future 8.No role in the prevention of emergence of new variant		Against
15	Vaccinating children against COVID-19: maximize uptake among adults while prioritizing the most vulnerable (29).	EliaAbi-Jaoude (2021)	Letter		1.Low risk of infection among children 2.No role in the prevention of disease transmission in community or household, 3.Scarcity of vaccine		Against
16	Should we delay COVID-19 vaccination in children? (30)	Dominic Wilkinson et al. (2021)	Head-to-head		1.Mild disease among children 2.Less cost-effectiveness ratio 3. Less cost-benefit ratio, 4.Unknown side effects, 5.No defense against the severe forms of COVID-19 infection 6.Safety problems, 7.The limited supply of vaccines, 8.No role in the prevention of COVID-19 transmission		Against
17	Should the UK vaccinate children and adolescents against COVID-19? (31)	Sonia Saxena et al. (2021)	Editorial	1.Helps children with school reopening 2.Stop emergence of new variants 3.Reduction in disruption of health care facility 41.Role in reduction of household transmission	1.Low disease burden 2.Less cost-effectiveness ratio, 3.Severe adverse events following vaccination 4.No defense against COVID-19 infection	1.Concerns about vaccine safety 2.Uncertain defense against long COVID	Against
18	COVID-19: cases in children rise sharply in the US as doctors call for vaccine approval (32).	Janice Hopkins Tanne (2021)	News	1.Increasing disease burden among children 2.Less number of severe adverse events 3.Stop the emergence of a new virus variant			Favor
19	Other good reasons for COVID-19 vaccination in pre-adolescent and adolescent populations (33).	Federico Marchetti et al. (2021)	Letter	1.Restoration of normal social life and school activities, 2.Favorable risk-benefit ratio 3.Helps in the development of Herd immunity 1.Improving the mental health of the adolescent	1.Direct cost of vaccination is high	1.Uncertain about severe adverse events 2.Uncertain about prevention of community transmission	Favor
20	Inviting adolescents aged 12-17 for COVID-19 vaccination: the need for patience (34).	Peter A M de Beer et al. (2021)	Letter	1.Reduce MIS-C among children, 2.Defense against long COVID			Favor
21	COVID-19 vaccines for teenagers: conversations and consent (35).	Sonia Saxena et al. (2021)	Editorial	1.Less severe adverse events following vaccination 2.Helpful for school reopening	1.Less disease burden 2.No role in the development of herd immunity in the community 3.Low rate of infection among children, 4.Mild form of the disease among children 5.Difficult consent process for minors, 6. High vaccine hesitancy	1.Uncertain risk-benefit ratio	Against
22	COVID-19 vaccines for children and adolescents in Africa: aligning our priorities to situational realities (36).	Kaymarlin Govender et al. (2022)	Analysis	1.Increasing disease burden and hospitalization rate among children, 2. Provide defense against COVID-19 infection 3.Protect against the severe form of COVID-19 4.The vaccine reduces the chances of mortality, 5.Reduce educational disruption, 6.Will improve routine vaccine delivery infrastructure, 7.Good safety and effectiveness of the vaccine 8.Faster return to prepandemic activity and economic stability 9.Stop emergence of new vaccine variant	1.Mild infection among children 2.Increasing vaccine hesitancy 3.High level of vaccine inequality 4.Uncertain role in the reduction of community transmission		Favor
23	Vaccinating adolescents wisely against COVID-19 (37).	Maurizio Bonati et al. (2021)	Editorial	1.Provide defense against COVID-19 infection 2.Reduces the severity of disease, 3.Reduce fear of COVID-19 among children and parents 4.Few severe adverse events	1.Not helpful in school reopening 2.Overall general population risk-benefit is less 3.Mild symptoms among children 4.The direct risk-benefit ratio is less		Both or uncertain
24	The Importance of Advancing COVID-19 Vaccines in Children (38).	Carol M. Kao et al. (2022)	Review	1.Increasing disease burden among children, 2.Provide defense against the severe forms of COVID-19 3.Reduces chances of community transmission, 4.Presence of a functional children's universal immunization infrastructure.			Favor
25	Warp speed for coronavirus disease 2019 (COVID-19) vaccines: why are children stuck in neutral? (39)	Evan J. Anderson et al. (2021)	Viewpoint	1.Vaccination will reduce the disease burden, MIS-C, and hospitalization rate among children, 2.The vaccine is safe and less adverse events, 3. School reopening, 4.Improve the mental health status of the parents	1.Disease is in mild form among children	1.Uncertain role in the prevention of community transmission,	Favor
26	COVID-19 vaccine for children in China: when to start? Mandatory or voluntary? (40)	Fei Liu et al. (2021)	Correspondence	1.Increasing disease burden among children, 2.Favorable Safety data	1.High vaccine hesitancy 2.Less data on vaccine effectiveness	1.Uncertain role in the prevention of disease transmission	Both or uncertain
27	Should children get COVID-19 vaccines? What the science says (41).	Heidi Ledford (2021)	News on focus	1.Prevent the possible emergence of a new variant of COVID-19 2. Protection against Co-infection of COVID-19 and other circulating routine viruses 3.Role in development of Herd immunity 4.Useful for high-risk groups of children	1.Severity is less among children,	1.Uncertain about disease burden among children 2.Uncertain about the result of safety trial of vaccines among children 3.Uncertain about the adverse events following vaccination 4.Uncertain about the role in the prevention of COVID transmission 5.Long-term safety of the vaccine unknown	Both or uncertain
28	COVID-19 vaccination in children and university students (42).	John P. A. Ioannidis (2021)	Commentary	1.Early normality like prepandemic period, 2.Severity of the disease is high among children 3.High risk-benefit ratio, 4.Reduce disease burden, severity, and mortality, 5.Fewer chances of serious adverse events and long-term events 6.Good defense against COVID−19 infection 7. Helps in school reopening 8.The epidemiological shift of disease in higher age groups without vaccination 9.Indirect protection for elderly	1.Long-term safety unknown, 2.Less duration of protection, 3. Large level of the population is already exposed 4.Socio-economic inequality in vaccine delivery	1.Uncertain protection from reinfection 2.Uncertain role of protection against the new variant 3.Uncertain role in the prevention of long COVID 4.Uncertain role in the prevention of community transmission 5.Uncertain about vaccine safety and efficacy	Both or uncertain
29	COVID-19 vaccination in children and adolescents—a joint statement of the European Academy of Pediatrics and the European Confederation for Primary Care Pediatricians (43).	ŁukaszDembin' ski et al. (2021)	Perspective	1.School reopening easier 2.Enhance herd immunity 3.Improve the mental health status of children and parents 4.Prevent interruption of health care facilities 5.Less vaccine hesitancy	1.Low disease burden and severity among children 2.Ethical concerns of minor vaccination,	1.Uncertain about the result of clinical trials on vaccine efficacy, and safety among children 2.Uncertain about adverse events of vaccine 3. Uncertain role in protection against MIS-C 4.Uncertain protection against long COVID-19 5.Uncertain role in the prevention of community transmission,	Both or uncertain
30	Pediatric off-label use of COVID-19 vaccines: ethical and legal considerations (44).	Elizabeth lanphier et al. (2021)	Report		1.Ethical and legal concerns of “off-level” COVID-19 vaccine for children		Against
31	Should we mandate a COVID-19 vaccine for children? (45)	Douglas J. Opel et al. (2020)	Editorial	1.High disease burden 2.Have a role in developing herd immunity	1.Mild infection among children, 2.No role in the prevention of disease transmission	1.Uncertain about vaccine safety and efficacy 2.Uncertain about the risk-benefit ratio	Uncertain
32	COVID-19 and routine childhood vaccinations—identifying gaps and informing solutions (46).	Brian P. Jenssen et al. (2022)	Opinion		1.Interruption of routine childhood immunization		Against
33	COVID-19 vaccination of minors without parental consent (47).	Larissa Morgan et al. (2021)	Viewpoint	1.Increasing disease burden among children	1.Milder form of the disease among children 2.Vaccine hesitancy among community 3.Difficult Consent process for vaccination among minors	1.Uncertain role in the prevention of disease transmission	Against
34	Increased incidence of COVID-19 in younger patients (May–July 2021)—an argument for extending vaccination? (48)	Paul W. Bird et al. (2021)	Letter to Editor	1.Increasing disease burden among children, 2.The increasing rate of hospitalization among children	1.Breakthrough infection possible 2.Vaccine hesitancy a problem	1.Uncertain duration of protection	Favor
35	Should universities mandate the COVID-19 vaccine? (49)	Constance Burke (2021)	Viewpoint	1.Less Vaccine hesitancy	1.Ethical issues of adolescent vaccination, 2.Concerns related to vaccine supply 3.Failure of compulsory vaccination laws in the past		Against
36	Impact of COVID-19 on women and children and the need for a gendered approach in vaccine development (50).	Kranti Suresh Vora et al. (2020)	Mini review	1.The gender-based difference in COVID-19 vaccine development			Favor
37	Considerations for mandating a new COVID-19 vaccine in the USA for children and adults (51).	Dorit R. Reisset al. (2020)	Essay	1.Good safety and effectiveness of the vaccine 2.Less number of serious adverse events 3.Faster return to prepandemic activity 4. Essential for high-risk group children	1.Ethical concerns, legal issues, and political factors related to children's COVID-19 vaccinations		Against
38	We should not vaccinate the young to protect the old: a response to Giubilini, Savulescu, andWilkinson (52).	Iñigode Miguel Beriain (2020)	Commentary		1.Mild infection among children 2.Severe adverse events possible 3.Ethical concerns, 4.Lack of scientific evidence of benefits among children vaccination, 5.Unknown risk among children 6.No role in the reduction of disease transmission	1.Uncertain vaccine efficacy and safety	Against
39	Zero-sum or worse? Considering detrimental effects of selective mandates on voluntary childhood vaccinations (53).	Philipp Sprengholz (2021)	Letter to Editor		1.Mild disease among children 2.Parental hesitancy and anger	1.Uncertain vaccine efficacy and effectiveness	Against
40	Mass infection is not an option: we must do more to protect our young (54).	Deepti Gurdasani et al. (2021)	Correspondence	1.Increasing disease burden among unvaccinated children 2.Prevention of educational disruption, 3.Prevent the emergence of new vaccine resistance variance, 4.Reduce disruption of health care facilities			Favor
41	COVID-19 vaccines for children in LMICs: another equity issue (55).	Beate Kampmann et al. (2021)	Comment	1.High rate of COVID-19 infected children in Low- and middle-income countries 2.Reduce disruption of routine vaccination services among children, 3.Improve the psychological health of the children after lockdown 4.Beneficial for the high-risk groups of children	1.Mild form of the disease among children 2.Vaccine inequality	1.Uncertain vaccine efficacy and safety 2.Uncertain role of the vaccine in school reopening	Both or uncertain
42	Herd immunity and vaccination of children for COVID-19 (56)	Thirumalaisamy P. Velavana et al. (2020)	Editorial	1.Good vaccine safety and efficacy 2.If children had reached herd immunity by vaccination it will prevent the transmission of disease to others,	1.Mild form of COVID 2.Low rate of infection	1.Uncertain about serious adverse events 2.Uncertain about protective role against preventing MIS-C 3.Uncertain about the role of the vaccine in the prevention of household transmission	Both or uncertain
43	Pediatric vaccination against COVID-19 and despite COVID-19 (57).	Federico Martinón-Torres (2020)	Editorial	1.Provide protection against COVID-19 infection 2.Prevention of post-COVID sequelae, 3.Good safety of the vaccine, 4.Protection against new variants 5.Provide defense against long COVID-19	1.Low disease burden among children 2.Mild infection among children 3.The problem of ethical consideration	1.Uncertain about severe adverse events 2.Uncertain about a faster return to pre-pandemic activity 3.Uncertain about the role of school reopening 4.Uncertain about protective role against PIMS/MIS-C 5. Doubtful in lowering the transmission 6.Uncertain about indirect harms of lockdown 7.Uncertain about indirect benefits toward elderly from childhood vaccination	Both or uncertain
44	COVID-19 vaccines for children younger than 12 years: are we ready? (58)	XiaohuiZou, Bin Cao (2021)	Comment	1.High disease burden 2.Provide protection against COVID−19 infection 3.Prevention of severe forms of COVID 4.Helpful in the development of herd immunity 5.Prevention of MIS-C	1.Mild infection among children 2.A large number of the population has already been exposed and developed natural immunity against the virus	1.Uncertain safety, tolerability, efficacy, and immunogenicity in youths, 2.Duration of protection by the vaccine is uncertain 3.Unknown role in the prevention of transmission	Both or uncertain
45	Global ethical considerations regarding mandatory vaccination in children (59).	Julian S, et al. (2021)	Reflection	1.Protect against the severe form of COVID	1.Mild infection among children 2.Long-term safety unknown 3. Severe adverse events possible 4.Ethical issues/difficulties regarding mandatory vaccination 5.No role in the reduction of community transmission		Against
46	Voluntary COVID-19 vaccination of children: a social responsibility (60).	Brusa M, et al. (2021)	Clinical ethics	1.High disease burden among children 2.Vaccines is having good safety and efficacy trial 3.Less number of severe adverse events following vaccination 4.Provide protection against COVID-19 infection 5. Protection against the severe form of COVID-19 6.Early reopening of school for children 7.Favorable risk-benefit ratio 8.Improve the mental health status of children 9.Beneficial for the high-risk group of children 10.Ethical issues (autonomy, justice, global justice,) social responsibility in Favor of children's vaccination			Favor
47	Vaccinating children against COVID-19—the lessons of measles (61).	Klass P, et al. (2021)	Perspective	1.Good protection against COVID-19 infection among children 2.Provide protection against the severe form of COVID−19 1.Early back to normal life for children 3.Faster return to pre-pandemic level in economic activity 4.Beneficial role in developing herd immunity 5. Improve the social interaction and mental health status of the children 6. Protecting children against COVID-19 infection is both an ethical obligation and a practical necessity 7.Role in reduction of household transmission of COVID-19 8.Will reduce the indirect harm of lockdown			Favor
48	Adolescents, parents, and COVID-19 vaccination—who should decide? (62)	McGrew S et al. (2022)	Perspective	1.Helps in early school reopening 2.Improve the mental health status of the children	1.Mild disease among children 2.Ethical issues/ difficulties of consent in adolescent COVID vaccination		Favor
49	Is there a role for childhood vaccination against COVID-19? (63)	Eberhardt CS, et al. (2020)	Review		1.Low incidence and disease severity, 2.Less protection against the severe form of COVID-19 3.Negative effects on routine childhood vaccination	1.Unknown safety and efficacy among children, 2.Uncertain role in the prevention of COVID-19 transmission among household	Against
50	Considering mandatory vaccination of children for COVID-19 (64).	Plotkin SA, et al. (2021)	Perspective	1.Increasing disease burden and mortality among children, 2.Faster return to prepandemic activity 3.Severe infection and MIS-C are also occurring among children 4.Role in household and community transmission 5.At least a minimal level of protection against COVID-19 6.Effective in reaching herd immunity 7.High successes record of childhood vaccination in the past 8.A well-developed infrastructure for childhood vaccination 9.School reopening 10.Prevent the emergence of a new variant of the virus	1.Asymptomatic or mild infection among children		Favor
51	How can we best use COVID-19 vaccines in adolescents? An international perspective (65).	Youjia Zhong et al. (2021)	Commentary	1.Provide defense against the severe form of COVID-19 2.Favorable Risk-benefit analysis 3.Favorable role in developing herd immunity against COVID-19 4.Necessary for the high-risk group of children 5.No ethical problem with children's vaccination against COVID-19	1.Majority of mild infections among children	1.Unknown safety and efficacy of the vaccine 2.Uncertain protective role against PIMS	Favor
52	How can we best use COVID-19 vaccines in adolescents? A perspective from the United States (66).	Donna L. Tyungu et al. (2021)	Commentary	1.Increasing disease burden among children 2.Provide protection against COVID-19 infection among children 3.Good defense against the severe form of COVID-19 or complication 4.Helps in school reopening 5.Faster return to pre-pandemic activity 6.Favorable Risk-benefit ratio 7.Stop emergence of new vaccine variant 8.Protection against PIMS/MIS-C among children 9.Good defense against long COVID-19 10.Have a role in the reduction of community transmission	1.Limited COVID-19 vaccine supply	1.Uncertain about the severe adverse events of the vaccine	Favor
53	COVID-19 and vaccination of children and adolescents: prospects and challenges (67).	Gregory D, et al. (2021)	Commentary	1.High disease burden among children 2.Provide protection against COVID-19 infection 3.Provide protection against COVID-19 complications 4.Favorable for inclusion in the immunization schedule	1.Low mortality and morbidity among children, 2.Less duration of protection 3.Ethical concerns in minor vaccine trail, 4.High vaccine hesitancy 5.Problem of vaccine inequality	1.Uncertain role in lowering community transmission of COVID-19	Uncertain
54	Viewpoint of the European pediatric societies over severe acute respiratory syndrome coronavirus 2 (COVID-19) vaccination in children younger than age 12 years amid return to school and the surging virus variants (68).	Massimo Pettoello-Mantovani (2021)	Viewpoint	1.Increase in the number of cases among children 2.Provide protection against COVID−19 infection 3.Helpful in reducing the severity of COVID-19 4.Definitive role in developing herd immunity in the community 5.Stop the emergence of a new virus variant 6.Increase in MIS-C among children		1.Concern about the safety, efficacy, and quality of vaccines among children	Favor
55	Education and mental health: good reasons to vaccinate children (69).	Simon Cauchemez et al. (2021)	Correspondence	1.Increasing disease burden among children 2.Vaccines having good safety and effectiveness among children 3.School reopening 4. Improvement of the mental health status of children	1.Problem of ethical issues related to minor vaccination	1.Uncertain about severe adverse events of the vaccine among children 2. Uncertain about prevention of transmission of the virus	Favor
56	Crossing the Rubicon: a fine line between waiting and vaccinating adolescents against COVID-19 (70).	Shamez N Ladhani et al. (2021)	Review	1.High disease burden 2.Direct protection against COVID 19 3. Beneficial for high-risk group children 41.Vaccines provide defense against long COVID 5.COVID vaccine has a protective role against PMIS-TS 6.Reduce hospitalization and disease burden 7.Improve the mental health status of children 8.Prevent community transmission of the virus 9.Prevent asymptomatic infection 10.Helps in school reopening	1.Mild disease among children 2.Possibility of severe adverse event 3.No role in the development of herd immunity among community 4.Not favorable risk-benefit ratio 5.High rate of adult vaccination will reduce the necessity for childhood COVID-19 vaccination	1.Limited data on population-level safety, efficacy,	Both or uncertain
57	COVID-19 herd immunity by immunization: are children in the herd? (71)	Stephen Obaro (2021)	Comment		1.Low level of infection among children 2.Vaccine efficacy and safety are questionable among children 3.No beneficial risk-benefit ratio for childhood COVID-19 vaccination 4.Less role in developing herd immunity 5.Inversely effect routine childhood immunization 6.Ethical issues related to risk-benefit among children 7.High rate of adult vaccination will reduce the necessity for childhood COVID-19 vaccination	1.Uncertain role in the prevention of household transmission of the virus	Against
58	Global Pediatric Pulmonology Alliance (GPPA) proposal for COVID-19 vaccination in children (72).	Lance E. Rodewald et al. (2021)	Editorial	1.Good efficacy and safety of the vaccine for children 2.Prevent COVID−19 infection among children 3.Prevent severe forms of COVID-19 infection 4.Beneficial role in the reduction of transmission of the virus in the community 5.Increasing disease burden among children, 6.New variant highly transmissible among children, 7.Increasing adult vaccination makes children vulnerable, 8.Indirect suffering of children from COVID-19 (becoming orphan, malnutrition among children, etc.) 9.Helpful for developing herd immunity	1.Mild disease among children		Favor
59	Recommendations for the urgent need to vaccinate school-aged and adolescent children against COVID-19 in the Asia–Pacific region (73).	Jun Kobayashi et al. (2021)	Letter to Editor	1.Increase risk of infection, 2.Vaccines is having good safety and efficacy in trail 3.Less adverse events following vaccination among children 4.Helpful in school reopening 5.Prevent the development of a new variant of the virus, 6.Reduces indirect harms of lockdown 7.Severe COVID-19 cases among children, 8.Have a definitive role in the reduction of household transmission of the virus			Favor
60	Children are the key to the Endgame: a case for routine pediatric COVID-19 vaccination (74).	Mark R. Schleiss et al. (2021)	Commentary	1.Case load is increasing, 2.Protecting role on long COVID-19 and MIS-C, 3.Severe side effects are very rare, 4.Prevent COVID-19 infection among children 5.Protective role against PIMS/MIS-C among children 6.Safety, efficacy, and effectiveness data among children are becoming available, 7.School reopening and normality like prepandemic period,			Favor
				8. A well-developed infrastructure for childhood vaccination will be helpful. 9.Improve the psychological status of the children following lockdown 10.Children's vaccination will reduce the necessity for the strict requirement of personal protective equipment (e.g.: masks) 11.Provide defense against long COVID-19 12.No ethical problems with children's vaccination			
61	COVID-19 vaccine for children: the challenge of making a decision (75).	María Elina Serra (2021)	Comment	1.Helps in developing herd immunity	1.Mild form of the disease among children 2.The problem of consent among children, 3.Vaccine safety and efficacy are not very effective	1.Uncertain role in the prevention of community transmission, 2. Uncertain vaccine hesitancy 3.Uncertain risk-benefit ratio of vaccine	Both or uncertain
62	Why it is important to develop an effective and safe pediatric COVID-19 vaccine (76).	Nicola Principi et al. (2021)	Viewpoint	1.Increasing caseload among children, 2.Vaccines is having good efficacy and effectiveness among children 3.Indirect benefit of children's vaccination will be for elderly 4.Helps in reduction of disruption of other health care services,		1.Uncertain role in protection against PIMS/MIS-C 21.Uncertain role of the vaccine in the prevention of household transmission,	Favor
63	COVID-19 vaccines for children (77).	Jeffrey S. Gerber et al. (2021)	Editorial	1.Increasing disease burden among children, 2.Resumption of pre-pandemic period activity among children 3.A well-developed infrastructure for childhood vaccination will be helpful	1.High Vaccine hesitancy, 2.Severe adverse is present among children following vaccination	1.Uncertain safety, efficacy, and effectives of the vaccine among children	Both or uncertain
64	Expert consensus on COVID-19 vaccination in children (78).	Yue-Jie Zheng et al. (2021)	Editorial	1.Increasing disease burden, 2.Helpful in developing herd immunity 3.Vaccine has a role in the prevention of community transmission, 4.The vaccine is safe and less adverse effects among children, 5.Stop emergence of new vaccine variant			Favor

**Figure 2:**
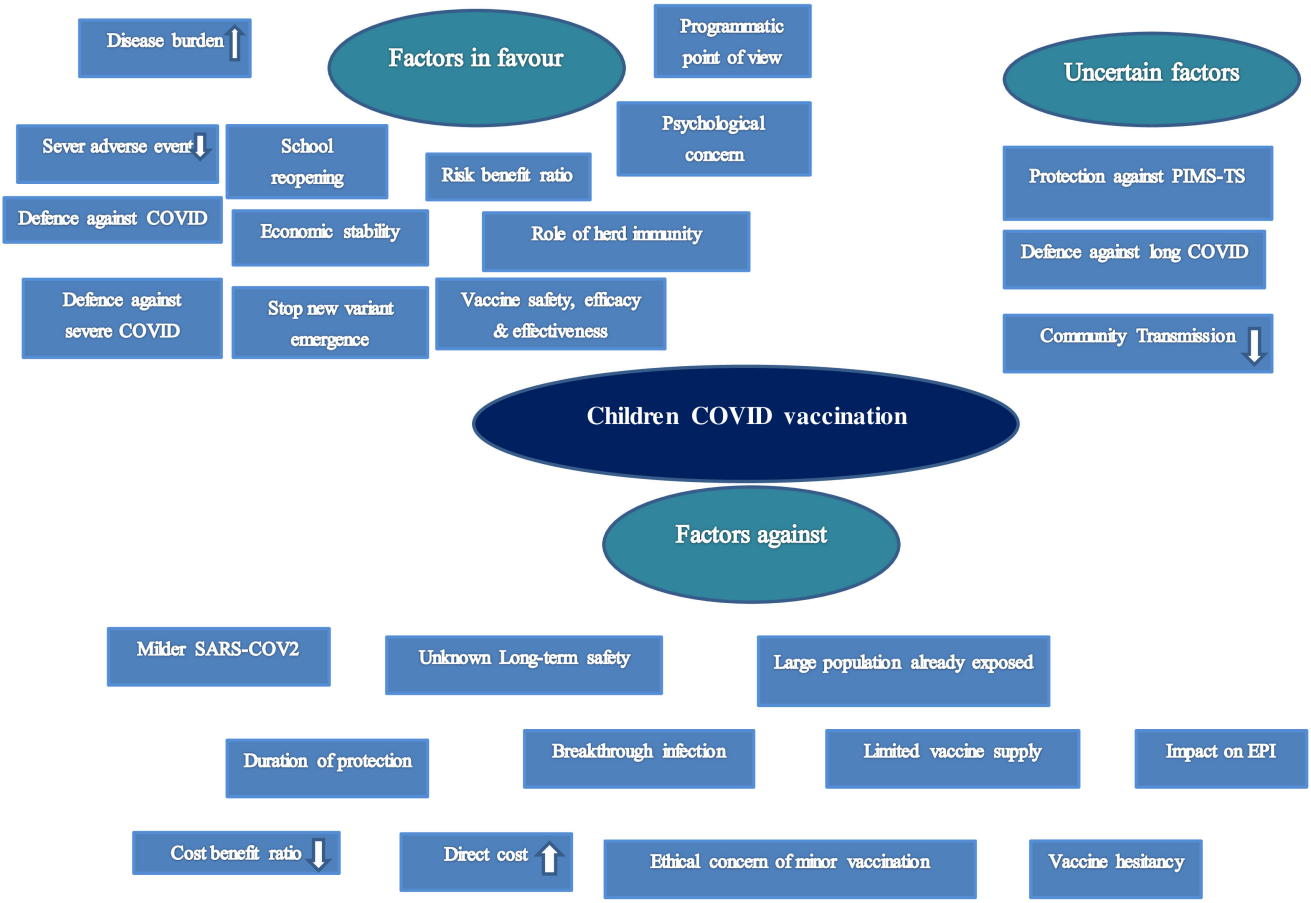
Factors to consider in relation to COVID-19 vaccination in children.

The total number of factors discussed in relation to childhood COVID-19 vaccination was: 41 (Figure 2).

(1) Disease burden among children: 41 articles

(2) Vaccine safety, efficacy, and effectiveness: 40 articles

(3) Level of severe adverse events: 31 articles

(4) Defense against COVID-19 infection: 26 articles

(5) Defense against the severe form of COVID: 23 articles

(6) Back to normal for children (school reopening): 26 articles

(7) Faster return to prepandemic activity and economic stability: 10 articles

(8) Stop the emergence of new variant: 17 articles

(9) Cost-benefit analysis: 5 articles

(10) Risk-benefit analysis: 18 articles

(11) Role of herd immunity: 17 articles

(12) Programmatic point of view: 10 articles

(13) Psychological aspects: 16 articles

(14) Social and ethical responsibility: 2 articles

(15) Role of PMIS/MIS-C: 16 articles

(16) Defense against long COVID-19: 13 articles

(17) Contribution in the community or household transmissions: 41 articles

(18) Children have milder COVID-19 Infection: 30 articles

(19) Long-term safety unknown: 7 articles

(20) The large population already exposed/immune: 2 articles

(21) Questionable duration of protection: 5

(22) Breakthrough infection: 2

(23) Limited vaccine supply: 5 articles

(24) Impact on routine immunization: 5 articles

(25) High direct cost of vaccination: 3 articles

(26) Ethical concern: 20 articles

(27) Vaccine hesitancy: 15 articles

(28) High-risk group of children requires COVID-19 vaccination: 9

(29) Problem of Orphanage, malnutrition among children: 1 article

(30) Decrease requirement of personal protective equipment: 1 article

(31) Indirect harm of lockdown: 5 articles

(32) Indirect benefit of childhood vaccination for elderly: 4 articles

(33) Reduction in disruption of routine healthcare facility: 7 articles

(34) Epidemiological shift in higher age group children: 1

(35) Beneficial for international travel: 1 article

(36) Higher case fatality and low transmission rate in future: 1 article

(37) High rate of adult vaccination will reduce the necessity of childhood vaccination: 2 articles

(38) Failure of compulsory vaccination law: 1 article

(39) Vaccine delivery inequality: 5 articles

(40) Gender-based inequality in the vaccine: 1 article

(41) Socio-economic inequality in vaccination: 1 article

Top three favorable factors for childhood COVID-19 vaccination mentioned by the highest number of articles:

(a) Increasing rate of disease burden (29 articles)
(b) Prevention of interruption of academic activities of children or school reopening: 24 articles
(c) Role in defense against COVID-19 infection (21 articles),

Top three unfavorable/against factors for childhood COVID-19 vaccination mentioned by the highest number of articles:

(a) Mild infection among children (27 articles),
(b) Ethical concerns and legal problems regarding the consent of minors (17 articles),
(c) Vaccine hesitancy among parents for childhood vaccination (11 articles).

Top three uncertain factors for childhood COVID-19 vaccination mentioned by the highest number of articles:

(a) Contribution in reduction of community or household transmissions: 19 articles
(b) Protective role against PMIS/MIS-C: 10 articles
(c) Defense against long COVID-19: 7 articles

## Discussion

### Factors in favor

#### Scientific evidence

##### Disease burden among children

Children of all ages may be infected with COVID-19, according to the evidence, although the disease burden is lower in them due to a lower prevalence of exposure and infrequent testing. Regardless of symptoms, studies on acute or previous COVID-19 infections in children and adolescents have shown that the infection incidence is comparable to that of adults. A high disease burden despite mild symptoms favors COVID-19 vaccination in children (21, 26, 27, 32, 36, 38, 48, 72, 77, 78). According to a multicentric surveillance study from many nations, the infection rate (laboratory-confirmed cases) among kids was as high as 18%. According to a CDC report, around 16–17% of laboratory-confirmed COVID-19 cases in the United States involved children. However, this percentage may be an underestimate of the true situation given the significant number of asymptomatic or mild cases among children that have not yet been tested (79). In total, 41 (64.0%) articles were discussing on the trend of COVID-19 in children, among them authors of 29 articles (45.3%) (16, 17, 21, 22, 26, 27, 32, 36, 38–40, 45, 47, 48, 54, 55, 58, 60, 64, 66–70, 73, 74, 76–78) were only highlighting high disease burden among children. Similarly, author of 10 articles (15.6%) (5, 19, 20, 28, 31, 35, 43, 57, 63, 71) was against it. Two of the articles (3.1%) (24, 41) focused on both the positive and negative aspects.

The infection incidence among youngsters could increase globally in future, like in South Africa or the United States, due to the advent of novel variations (e.g., Omicron, IHU, etc.) (48, 80).

##### Vaccine safety, efficacy, and effectiveness

Similar to the adult COVID-19 vaccine trial for the safety and efficacy of different COVID-19 vaccines, the children's trial, also in different age groups, has been conducted globally and data have been encouraging. Although World Health Organization (WHO) has approved the Pfizer-BioNTech (BNT162b2) vaccine and Moderna for children vaccination with an efficacy of 90.7% (5–11 years) and 100.0% (12–17 years), India's covaxine vaccine shows 95–98% efficacy (2–18 years) and Soberana 02 of Cuba (92.4%), Sinopharma, and Sinovac vaccine also shows a high level of safety and efficacy among children (9, 81–86). There are 18 studies (19, 21, 22, 26, 27, 36, 39, 40, 51, 56, 57, 60, 69, 72–74, 76, 78) which favor COVID vaccination in children in relation to safety, efficacy, and effectiveness, similarly 6 studies (5, 23, 30, 70, 71, 75) were raising concern about vaccine safety, efficacy, and effectiveness. And 16 studies were neutral or uncertain (16, 17, 31, 40–43, 45, 52, 53, 55, 58, 63, 65, 68, 77).

##### Level of severe adverse events

Among all the articles, 7 articles (30, 31, 40, 52, 59, 70, 77) were highlighting all adverse events as major events related with COVID vaccination, whereas 14 articles (19, 21, 22, 24, 26, 27, 32, 35, 39, 42, 51, 60, 73, 74) focusing all those events as minor or rare events; 10 articles (16, 17, 33, 41–43, 56, 57, 66, 69) were reflecting in a neutral tone about adverse events following COVID vaccination. Myocarditis (6 articles) (16, 17, 24, 31, 42, 58), pericarditis (6 articles) (16, 17, 24, 31, 42, 58). Chest pain (3 articles) [17, 21, 254], Fever (2 articles) (17, 21), Myalgia (2 articles) (17, 21), fatigue (2 articles) (17, 21), cerebral venous thrombosis (2 articles) (16, 17), MIS-C (2 articles) (16, 17), and headache, decreased left ventricular ejection, dysrhythmia, Vaccine-induced thrombocytopenia (VITT), Pediatric nephrotic syndrome, sore throat, and neck pain were reported by one article each. Myocarditis, which was reported and verified in 1,626 cases in the USA, was the most frequent adverse event associated with the COVID-19 vaccination in the 16- to 24-year-old age group (December 2020 to August 2021) (87).

##### Defense against COVID-19 infection

Evidence suggests that the rates of infection, as well as the severity of symptoms of COVID-19, are comparatively less among children, but the report of WHO has shown that from 30 December 2019 to 25 October 2021, COVID-related reported deaths were 1,797 among under 5 years, 1,328 among 5–14 years, and 7,023 among 15–24 years. It is also a fact that overlapping clinical presentation of COVID-19 with various childhood upper respiratory tract infections leads to misclassification as well as underestimation of COVID-19 in this age group (88). Authors have found that 21 articles (16, 17, 19, 21, 22, 26, 27, 36, 37, 42, 57, 58, 60, 61, 64, 66–68, 70, 72, 74) were pointing toward the role of COVID-19 vaccine in prevention of infection among children, whereas five articles (5, 20, 24, 30, 31) were not finding any significant role. The COVID-19 vaccine is having a definitive role in prevention among high-risk children (37, 89, 90). Countries having a high burden of different communicable diseases (e.g., malnutrition, tuberculosis, anemia, etc.) among children will face a serious impact unless universal COVID-19 vaccination is conducted for the high-risk group (90). Similarly countries having high population rates and a small proportion of infection will have a large number of absolute cases. Globally, primary immunization for different vaccine-preventable diseases (e.g., influenza, rubella, etc.) is conducted despite having a low incidence rate and hospitalization similar to the COVID-19 infection rate among children (88). Even adult emergency COVID-19 prophylactic vaccination was initiated in absence of any conclusive evidence.

##### Defense against COVID-19 complications (severe form of COVID-19)

The severe form of COVID-19 infection is usually seen among children with comorbidities, for example, malignancy, immunodeficiency, respiratory, cardiac, renal diseases, and so on (89, 91, 92). Even among healthy children also the chances of Multisystem inflammatory Syndrome of Childhood (MIS-C) (16, 43, 74), long COVID (43), severe COVID (43), critical illness (36), and increased duration of disease (43) are more in the absence of vaccination. Among the 64 articles, 19 articles (16, 19, 22, 36–39, 57–61, 65–68, 70, 72, 74) have emphasized the role of the COVID-19 vaccine against the severe form of disease, whereas only four articles (30, 31, 63) have highlighted no role in it including one uncertain article (24). With the emergence of a new variant of concern, the COVID risk of severe infection among healthy children might be many folds high in the future. Recently Omicron variant with more than 50 mutations has infected children under 5 years at a very high rate, especially in African countries (15).

##### Back to normal for children (e.g., school reopening)

In the first and second waves of COVID-19, school closure (partial/complete) was done as a presumptive approach rather than a risk-based approach (88). School closure had a significant negative impact on the educational services of the children, similarly, it had physical, mental, and emotional distress also (93). Although in the early part of the pandemic it was evident that school settings had a potential role in COVID-19 transmission, and when there is rampant community transmission of the virus, there is hardly any beneficial effect from the school closure (94). Although the decision on COVID-19 vaccination among children should be based on multiple factors (equity, availability, scientific evidence, susceptibility, etc.), universal vaccination of children against COVID-19 will reduce the fear among children as well as parents also (17, 26, 33, 39, 69, 73, 74). Among 64 articles, 24 articles (16, 17, 22, 24, 26, 27, 31, 33, 35, 36, 39, 42, 43, 54, 60–62, 64, 66, 69, 70, 73, 74, 77) had the opinion for childhood COVID-19 vaccination for early school reopening but three articles (37, 55, 57) were uncertain or against it. COVID-19 vaccination might be an essential requirement for international travel also (16).

##### Faster return to pre-pandemic activity and economic stability

Children are the main victims of COVID-19, which has had a direct impact on childhood education (24 million children are not in school, which is equivalent to a loss of US$10 trillion). In addition, it has left a long-lasting scar on society in the form of poverty, malnutrition, unemployment, food insecurity, and economic instability (88). Worldwide most countries have already vaccinated or started vaccinating their adult population. Many nations have begun immunizing youngsters against COVID. If children in low- and middle-income nations receive vaccinations, it will be simpler for any nation to return to its pre-pandemic status, which calls for the early continuation of all activities (16, 17, 77). This will facilitate the rapid recovery of the economy (57). In the present review, nine articles (16, 27, 36, 42, 51, 61, 64, 66, 74) have highlighted the role of early childhood COVID vaccination for faster return of pre-pandemic activity in society whereas only one article (57) had a different opinion.

##### Stop the emergence of new variants

Although there is no accepted theory regarding “How to stop the emergence of the new variant,” the best possible way to prevent the emergence of a new variant is to stop or minimize the spread of the virus (95). The generation of new mutant variations of the coronavirus is facilitated by its high rate of community transmission, and new variants (delta and omicron) have been identified to have a significant part in the rise in pediatric cases (17). Roll out of adult COVID vaccination in full swing will make the virus easily transmittable among vulnerable children, giving rise to a new mutant variant (54, 96). In total, 13 articles (17, 27, 31, 32, 36, 41, 54, 57, 64, 66, 68, 73, 78) in the present review have raised concern about the emergence of new variants among children in future in the absence of childhood COVID vaccination whereas four authors (16, 24, 28, 42) were not in full agreement with them.

##### Risk benefits analysis

The risk-benefit ratio of COVID vaccination is favorable, especially for those having comorbidities. However, a few cases of myocarditis with the mRNA vaccine create some doubt about the favorable risk-benefit ratio, but there is not any sufficient evidence of this (19, 22, 65, 66). Among adolescents, a study conducted in England also highlighted a favorable risk-benefit ratio unless the incidence of the case comes down very low (97). Out of the 64 articles, 8 articles (17, 19, 26, 27, 33, 42, 60, 66) were in favor of positive risk-benefit ratio, whereas 5 articles (20, 30, 37, 70, 71) were against it and 4 authors (35, 45, 65, 75) were uncertain.

##### Role of herd immunity

Universal youth and children COVID-19 vaccine will not only benefit the recipient directly but also aid in attaining herd immunity among all age groups. It will be especially helpful for people (such as the elderly) for whom direct immunization is occasionally not possible due to a variety of health difficulties. Earlier, it had been evidenced that immunization of children was much more beneficial (e.g., influenza, pneumococcal, and many other diseases) rather than the elderly. If childhood vaccination creates herd immunity, then it will act as a barrier for all age groups (43, 56, 75, 98). In this review, only 14 articles (27, 33, 41, 43, 45, 56, 58, 61, 64, 65, 68, 72, 75, 78) were highlighting the role of herd immunity related to childhood COVID vaccination and 3 articles (35, 70, 71) was against this opinion.

#### Programmatic point of view

If a benefit-risk assessment of children's vaccination shows that COVID-19 vaccination is beneficial for the country, the already existing infrastructure, logistics, and manpower for routine immunization will be a booster, mainly for developing countries. It has been observed that in many developing countries, lack of enough manpower is a major hindrance in delaying COVID vaccination (99). It will be relatively simpler to vaccinate every child quickly if the government adds COVID-19 vaccination to routine immunization, especially in low- and middle-income nations. In total, 10 articles (16, 21, 26, 36, 38, 55, 64, 67, 74, 77) in the present review were highlighting the programmatic favorable point of COVID vaccine inclusion through routine immunization among children.

#### Psychosocial point of view

Parents' mental health will always be disrupted in a family where parents are immunized but the children are not. Parents will be free of phobia if all children receive the COVID-19 vaccine (100–102). Similarly, the free movement of children will also improve their social interaction and social health among them. In our present review, 16 articles (17, 21, 22, 24, 27, 33, 37, 39, 43, 55, 60–62, 69, 70, 74) were highlighting the mental health issue of the parents and necessity of social interaction among children in favor of childhood vaccination.

#### Ethical obligation and practical necessity of children's COVID-19 vaccination

Globally, different countries had given emergency approval for the adult COVID vaccination program at a very early stage in the absence of many unanswered questions of efficacy, effectiveness, and so on (e.g., COVID-19 adult Vaccination was rolled out in India with COVAXINE with the report of Phase II trial data which showed good efficacy and safety as emergency approval, similarly COVAXINE children (2–18 years) vaccine has also got approval for emergency use based on Phase I/II trial) (102, 103). Question can be raised why not the same principle can be used for rolling out children's COVID-19 vaccination, rather than waiting for more evidence on vaccine effectiveness in the reduction of disease transmission or severity among children. It is a social and ethical responsibility and practical necessity to immunize children against COVID-19 (60, 61, 64, 76). Two articles (17, 61) were highlighting the ethical obligations and social responsibility of childhood COVID vaccination in the present review.

Other than these major factors, few of the articles have highlighted some other relevant favorable factors related to childhood COVID vaccination: Indirect harm of lockdown (16, 57, 61, 73), indirect benefit of vaccination (27, 42, 57, 76), reduction in disruption of normal healthcare services (21, 27, 31, 43, 54, 57, 76) protection for high-risk group of children (5, 17, 28, 41, 51, 55, 60, 65, 70), benefit in international travel (16), less children become orphan or malnutrated (72), and decrease requirement of mask or other personal protective equipments (74).

### Factors with uncertainty

#### Protection against PIMS-TS or MIS-C

Multisystem inflammatory syndrome (MIS-C) or Pediatric Inflammatory multisystem syndrome, temporally associated with COVID-19 (PIMS-TS) is one of the life-threatening complications of COVID-19 (104). There is still a lack of substantial evidence about the pathogenesis of the condition that whether it is a complication of the natural process of COVID infection among children or as a result of antigen-antibody reaction following COVID vaccination (16). The USA had reported more than 2,300 cases of MIS-C among children (5–11 years) since the inception of the pandemic. A few isolated cases of MIS-C have been reported in a study conducted in the USA among adolescents following COVID-19 vaccination but most of them were not life-threatening (105, 106). In the present review, 16 articles were discussing the role of MIS-C and COVID-19 whereas 6 articles (23, 34, 58, 66, 70, 74) were favoring its protective role and 10 articles (16, 17, 19, 24, 39, 43, 56, 57, 65, 76) were uncertain about it.

#### Defense against long COVID-19

If symptoms of COVID-19 in children persist for 4 to 16 weeks or longer, then it is called long COVID-19 (97). Several studies report a prevalence of long COVID-19 ranging from 1.2 to 66%. But most of the studies might have the limitation of overestimation of the risk. Some studies have evidence about the protective effect of COVID-19 vaccination against long COVID-19 among adults but lack evidence in the case of children. So, vaccination for defense against long COVID-19 is a matter of debate (16, 17, 23, 107–109). In the present study, out of the 64 articles, 7 articles (16, 17, 19, 24, 31, 42, 43) were uncertain about the COVID vaccine's role in preventing long COVID-19 whereas 6 articles (26, 34, 57, 66, 70, 74) were completely favoring it.

#### Contribution to reducing community or household transmissions

Theory about community or household transmission of coronavirus is ever-evolving. In the early part of the infection, adults and the elderly were the sources of infection, whereas later, children were contributing a major role in transmission. Evidence also suggests that though no vaccine can give complete protection, the vaccinated individual has a lower rate of infection compared to the unvaccinated which establishes the efficacy level of the vaccine. But still, there is no conclusive evidence on COVID-19 vaccine's role in reducing transmission (community or household) (17, 28, 31, 42, 56, 58, 67, 74, 110, 111). About 17 articles (5, 17, 19, 21, 23, 31, 33, 38, 47, 61, 66, 67, 69, 70, 72, 73, 78) in present review were in favor of beneficial role of COVID vaccine in reducing childhood transmission, whereas 23 articles (16, 20, 22, 24, 28–30, 36, 39–43, 45, 52, 56–59, 63, 71, 75, 76) were uncertain or against the role of children in COVID transmission or beneficial role of COVID vaccine.

### Factors against

#### Children have milder COVID-19 infection

Studies suggest that children are less frequently infected by the COVID-19 virus and there are multiple factors/theories behind it. Frequent viral infections with other types of coronaviruses in the past, fewer ACE receptors or high activity of the ACE enzyme, well-developed thymus gland that produce very good T-cell and memory cell immunity, lower prevalence of comorbidity, high level of primary immunity by childhood vaccination, or cross-protection with BCG vaccination may be some of the factors behind it (1, 100, 112–115). In the present review, 27 articles (5, 16, 26–30, 35–37, 39, 41, 43, 45, 47, 52, 53, 55–59, 62, 65, 70, 72, 75) were highlighting the mild form of COVID-19 infection among children which prevents it from universal childhood COVID vaccination, whereas only 3 articles (42, 48, 64) were against it. However, Pulmonary embolism, myocarditis and cardiomyopathy, venous thromboembolism, acute or unspecific renal failure, Coagulation or hemorrhagic disorders, cardiac dysrhythmia, encephalopathy, and febrile seizures are some of the notable systemic complications of COVID among children (116–118).

#### Long-term safety unknown

There is a scarcity of clinical trials among children globally regarding the long-term as well as midterm adverse effects, whereas any kind of adverse event can produce a lifelong impact on children (23, 30, 31, 41, 64). mRNA COVID vaccine can cause genetic changes and anaphylaxis, and damage to vascular endothelial cells can exuberate lung and cardiovascular injury also. There is the possibility of myocardial fibrosis, cardiac dysfunction, and so on, following COVID-19 vaccination (16). In the present review, 4 articles (16, 42, 52, 59) was raising unknown long-term safety of COVID vaccine among children as a major negative factor for childhood COVID-19 vaccination whereas 3 articles (17, 24, 40) were uncertain about it.

#### A large population already exposed/immune

Globally, most countries have done complete/partial COVID vaccination of a major portion of the adult or elderly population. Seroprevalence studies also highlight that a major proportion of the population in different countries shows a high level of exposure to coronavirus (e.g., ICMR study of servo-prevalence in India during June–July 2021 was 67.6% and among them, 57.2% were 6–9 years age group, 68% in Estonia in mid-June 2021, and 59% among the unvaccinated in Poland in May 2021) (42, 119). So, if a large portion of the population has been already exposed/immunized or developed natural immunity, then there is less chance of infection, transmission, and severity of COVID among children. In the present review, two articles (42, 58) were highlighting it as a major negative factor for childhood COVID vaccination.

#### Duration of protection

A report by CDC has highlighted that immunological protection of the COVID vaccine declines 6–8 months following vaccination and different studies have also raised concern about the long effectiveness of the COVID vaccine (120). Since there is uncertainty about the long-term protective effect of the COVID-19 vaccine, exposing the children will not be a wise decision. Three articles (28, 42, 68) in this present review have shown negative concern about the duration of protection following COVID vaccination among children, whereas two were uncertain about it (48, 58).

#### Breakthrough infection

As the number of COVID immunized populations is increasing globally, the number of cases of reinfection or breakthrough infection also will raise because no vaccine can give complete protection. A report from Johns Hopkins has highlighted that 1 in 5,000 was the rate of reinfection/breakthrough infection in Washington state (17 January to 21 August 2021) among the fully vaccinated population. In that study, some areas had a breakthrough infection rate of 1 in 100 (121). Moreover, universal COVID-19 vaccinations for children will be a threat, as a newer variant of COVID-19 increases the chances of more breakthrough infections. In the present review, only two articles (42, 48) have raised concern about the relation between breakthrough infection and COVID vaccination among children.

#### Limited vaccine supply

One of the critical factors in deciding on universal COVID vaccination is the free supply and availability of the vaccine which is mainly lacking in low- and middle-income countries. Because of this scarcity of vaccines, 1 in 100 in low-income countries and 1 in 10 in lower-middle-income countries have achieved full vaccination status, whereas it is 1 in 2 in high-income countries (122). Therefore, constrain of the COVID-19 vaccine have forced many countries globally to follow a stepwise approach to COVID vaccination considering the risk factors (e.g., disease incidence, comorbidity, mortality, etc.) (30). Five articles (16, 29, 30, 49, 66) have considered limited vaccine supply as one of the important hindrances to universal COVID vaccination for children in the present review.

#### Impact on routine immunization

The COVID-19 pandemic has caused a huge disruption of the healthcare delivery services since the beginning of the pandemic and COVID vaccination also caused extra stress on the already overburdened healthcare system which lacks sufficient manpower, resources, and logistics, especially for lower economic countries (46, 55, 63, 71, 123). It has been reflected by a report of UNICEF that in 2020 number of missed doses of routine vaccines was the highest globally since 2009 (123). Condition is much worse in developing countries [e.g., In India, BCG and Pentavalent missed doses were 1 lakh and 2 lakh, respectively, in March 2020 (124), and in Pakistan, those who missed a dose of measles and polio were 40 and 50 million, respectively, during the same period] (125). Five articles (16, 17, 46, 63, 71) in the present review have shown the disruption of routine immunization or other health services as one of the negative factors/hindrances for universal childhood COVID vaccination.

#### Direct cost

Children's hospital admission and treatment costs for COVID-19 will be lower because hospitalization rates are so low, but their universal COVID vaccine will come at a significant direct cost. About three articles have highlighted it as a major negative factor for COVID vaccination among children (16, 20, 33).

#### Cost-benefit analysis

If we analyze direct cost vs. vaccine cost, then vaccine cost is cheaper than direct cost, for example, hospital admission and treatment cost, similarly if we consider the total cost (direct and indirect cost) vs. vaccine cost, then vaccine cost is much cheaper than the total cost that includes hospital admission and treatment cost, physical and mental health problems of children, mental health problems of parents, and so on (126).

A combined report of the World Bank, UNESCO, and UNICEF (December 2021) highlighted that the disruption of education among students will cause a $17 trillion loss of lifetime earnings, whereas a revised report has shown that the impact will be much higher (127). Therefore, if we talk about cost, then it is beneficial to vaccinate children against COVID-19. Vaccine cost is very less but the overall cost that will be paid in comparison to vaccination is much higher. In this review, one article (16) was highlighting a positive cost-benefit ratio for childhood vaccination, whereas four articles' (20, 28, 30, 31) opinions were vice-versa probably considering the caseload among children which will impact the cost-effectiveness of the program.

#### Ethical issues and concerns

Several ethical factors or concerns related to childhood COVID-19 vaccination have been highlighted by the articles included in the present review. A total of 17 articles were raising the direct ethical problem of minor or adolescent vaccination (17, 18, 20, 35, 43, 44, 47, 49, 51, 52, 57, 59, 62, 67, 69, 71, 75), whereas 3 articles (25, 65, 74) were either against it or uncertain. The difficult process of consent was highlighted by two articles (19, 35), violation of four principles of ethics was highlighted by one article (60), and the ethical problem of “off level vaccine use” (44), the legal problem of childhood vaccination (51), ethical problems of mandatory COVID vaccination (59), ethical problems of vaccine trail among minor (67), and ethical concern about risk-benefit ratio (71) were highlighted in one article each.

#### Vaccine hesitancy

According to WHO, vaccine hesitancy is among top 10 threats to global health. Vaccine hesitancy is a universal phenomenon. For children, especially with the new vaccine, it is very difficult to assure and obtain consent from parents. Sometimes one parent may agree, but others not. Parents are worried about side effects, the necessity of vaccination for their children, and the safety and efficacy of vaccines. Since only few vaccines are approved by the WHO while others are in the trial phase, on ethical grounds also, it is very difficult to vaccinate children against COVID-19 (19, 25, 35, 40, 44, 47, 49, 51–53, 59, 62, 68). In total, 11 articles (17, 18, 25, 27, 35, 36, 47, 48, 53, 67, 77) were highlighting the problem of vaccine hesitancy in this review.

#### Problems that need more concern as compared to COVID-19 vaccination

Developing and underdeveloped countries have the double burden of different communicable (e.g., malaria, tuberculosis, ARI, etc.) as well as non-communicable diseases (e.g., malnutrition) where the effect of these conditions may be more devastating than COVID. If a large number of resources (money, manpower) have been utilized for universal COVID vaccination, then it raises a serious question on the principle of equity and justice (128). However, only one article (71) was addressing this important issue of judicial distribution of scarce resources.

Similarly, an epidemiological shift in the age group following childhood vaccination (28, 42), vaccine inequality (24, 36, 55, 67), gender-based inequality in COVID vaccination (50), higher case fatality and low infection rate among children in future (28), high rates of adult vaccination can reduce the requirement of childhood vaccination (70, 71), failure of compulsory vaccination law in past (49), and socio-economic status based inequility (42) are some of the minor factors against childhood COVID vaccination highlighted by a few of the articles.

#### Age-related difference in the immune response to COVID-19 infection between children and adult

Moreover, a weak “Immunosenescence,” a mechanism developed from “inflamm-aging,” is associated with an age-related reduction in innate and adaptive immune response which leads to a reduced ability to fight with novel COVID-19 virus (129). Children have a high level of innate immunity, adaptive immunity, heterogeneous immunity, and off-target effect of vaccine which in turn develop a difference in the age-related immune response to COVID-19 infection (130).

#### Age-related difference in the immune response to COVID vaccine between children and adult

Similar to age-related decline in immune response following COVID infection, vaccine-induced immunity also followed the same trend and it had been found in the case of earlier vaccines for Hepatitis B, pneumococcal vaccine, and influenza (131). BioNTech/Pfizer BNT162b2 also found an inverse relationship between age- and vaccine-induced immunity (132). Moreover, Children's immune response varies significantly with age. The level of antigen, as well as dose, can vary for infants and toddlers compared to school-age children or adolescents. It also holds for booster dose which requires more clinical evidence (76).

## Conclusion

Regardless of whether children should receive the COVID-19 vaccine, many nations have already started immunizing kids in stages. Although there is a lack of detailed information regarding hospitalization, severity, and child COVID mortality, little study has been done on the COVID-19 vaccine's safety and long-term efficacy in children. The vaccination studies for COVID-19 aim to promote immunity and offer individual disease protection. Only a selected few COVID-19 vaccines have received emergency approval for use in children's vaccination, but they still need to demonstrate long-term efficacy in stopping or reducing coronavirus transmission. The role of COVID-19 vaccines in the prevention of disease or suppression of the severity of the disease is a question of debate.

Several criteria, including the role of children in transmission, vaccine safety, efficacy, and length of protection, will determine whether all children will eventually receive vaccinations. Most significantly, it will rely on whether such vaccines stop the spread of the disease, resulting in herd immunity for the community. Therefore, before being given to children, each vaccination should be properly evaluated and shown to be safe. The effectiveness of vaccination in preventing transmission has not yet been studied extensively (63). However, recently a study conducted in Singapore has highlighted the role of the BNT162b2 Vaccine in preventing the transmission of COVID-19 among children (133).

Nevertheless, the beneficial effect (e.g., good efficacy, reduced adverse events, safety, etc.) demonstrated by a few of the clinical trials on childhood COVID vaccination should be interpreted cautiously because of many methodological limitations (e.g., limited sample size) which might have drastically changed when large-scale mass children vaccination starts.

The present systematic review has found that there was a diverse opinion among the experts on the matter of childhood COVID vaccination. In this regard, two-fifth of the article's major or prominent tone/theme was in favor of COVID vaccination among children, whereas three-fifth of the tone of the article was against, uncertain, or inconclusive. Among all the factors discussed/highlighted by the different authors, major factors which were mostly in favor of childhood COVID vaccination was the increasing rate of disease burden, school reopening, and defense against COVID infection. Similarly, major factors against childhood vaccination highlighted by the majority of articles were the mild nature of infection among children, ethical concerns and legal problems regarding the consent of minors' vaccination, and vaccine hesitancy among parents for childhood vaccination. Whereas, the vaccine's role in the reduction of community transmission, the protective role against PMIS/MIS-C, and the defense against long COVID was a major factor of uncertainty among them.

According to WHO, when creating their COVID-19 immunization policies and programs, nations should take into account the individual and population advantages of immunizing children in their unique epidemiological and socioeconomic environment.

Therefore, before moving further with COVID-19 immunization for children, a careful approach will be necessary due to the difference in disease progression rate among various age clusters.

## Strengths

The present review article may be probably one of the few studies that have tried to generate evidence using a systematic review format based on the opinion of different experts/reviewers on the topic of childhood COVID-19 vaccination considering all the possible positive and negative factors. The finding may be helpful for decision/policymakers to develop policy on evidence-based research for COVID vaccination among children. It will also encourage the generation of new research ideas/horizons for addressing the uncertain factors related to childhood COVID vaccination in the future.

## Limitations

All the articles included in the present systematic review were either review articles or opinions or viewpoints and so on. There is not a single original article that has covered all the possible factors for consideration in childhood COVID-19 vaccination. Authors have not tried to identify the interrater agreement between them which can be a potential source of bias in the case of theme generation of an article. Several articles were excluded from the review because of the unavailability of full text which might have influenced the analysis and conclusion. Since all the articles were review articles or expert opinions, the assessment of the quality of the included articles was not possible using any standard systematic review assessment tool.

## Data availability statement

The original contributions presented in the study are included in the article/supplementary material, further inquiries can be directed to the corresponding author.

## Author contributions

SP conceptualized the study, data analysis, manuscript writing, and editing. CM contributed in the data extraction process, reviewing of articles, and manuscript editing. All authors contributed to the article and approved the submitted version.

## Conflict of interest

The authors declare that the research was conducted in the absence of any commercial or financial relationships that could be construed as a potential conflict of interest.

## Publisher's note

All claims expressed in this article are solely those of the authors and do not necessarily represent those of their affiliated organizations, or those of the publisher, the editors and the reviewers. Any product that may be evaluated in this article, or claim that may be made by its manufacturer, is not guaranteed or endorsed by the publisher.
